# Bacterial pathogens and antimicrobial susceptibility in ocular infections: A study at Boru-Meda General Hospital, Dessie, Ethiopia

**DOI:** 10.1186/s12886-024-03544-0

**Published:** 2024-08-13

**Authors:** Tegegne Asfaw, Yeshi Metaferia, Endalkachew Gebretsadik Weldehanna, Daniel Gebretsadik Weldehanna

**Affiliations:** 1 Boru-meda General Hospital, Dessie, Ethiopia; 2 Department of Medical Laboratory Science, College of Medicine and Health Sciences, Wollo University, P.O. Box 1145, Dessie, Ethiopia; 3 Wastewater treatment plant laboratory, CGC Overseas Construction Group, P.O. Box 24529/100, Addis Ababa, Ethiopia

**Keywords:** Ocular infection, Peri-ocular infection, Bacterial profile, Antimicrobial susceptibility, Boru-meda general hospital, Dessie, Ethiopia

## Abstract

**Introduction:**

The eye consists of both internal and external compartments. Several variables, including microbes, dust, and high temperatures can cause eye illnesses that can result in blindness. Bacterial eye infections continue to be a major cause of ocular morbidity and blindness, and their prevalence is periodically rising. The objective of the study was to detect bacterial pathogens and assess their susceptibility profiles to antibiotics in the ophthalmology unit of Boru-meda Hospital in Dessie, Ethiopia.

**Methods:**

A hospital-based cross-sectional study was conducted from February 1 to April 30, 2021, among 319 study participants with symptomatic ocular or peri-ocular infections who were enrolled using a consecutive sampling technique. After proper specimen collection, the specimen was immediately inoculated with chocolate, blood, and MacConkey agar. After pure colonies were obtained, they were identified using standard microbiological methods. The Kirby Bauer disk diffusion method was used to test antimicrobial susceptibility patterns, based on the guidelines of the Clinical and Laboratory Standards Institute.

**Results:**

The majority of participants developed conjunctivitis 126 (39.5%), followed by blepharitis 47 (14.73%), and dacryocystitis 45 (14.1%). Overall, 164 (51.4%) participants were culture positive, six (1.9%) participants had mixed bacterial isolates, giving a total of 170 bacterial isolates with an isolation rate of 53.3%. The predominant species was CoNS 47 (27.6%), followed by *S. aureus* 38 (22.4%) and *Moraxella species* 32 (18.8%). The overall Multi-Drug Resistance (MDR) rate was 62.9%, with 33 (44.6%) being gram-negative and 74 (77.1%) being gram-positive isolates.

**Conclusion:**

Conjunctivitis was the dominant clinical case and *CoNS, was* the predominant isolate. A higher rate of MDR isolates, particularly gram-positive ones, was observed. Efficient peri-ocular or ocular bacterial infection surveillance, including microbiological laboratory data, is necessary for monitoring disease trends.

**Supplementary Information:**

The online version contains supplementary material available at 10.1186/s12886-024-03544-0.

## Introduction

The eye is a sensory organ used by humans and is essential for daily life. Sustainable Development Goals (SDGs) cannot be attained without eye health, and universal health coverage cannot exist without it [1]. The structure of the eye consists of two compartments: one internal and one external [2]. Sterilization is maintained in the interior compartment, which is physically isolated from the immune system. However, the exterior compartment of the eye is prone to contamination by potentially harmful microorganisms owing to exposure to the external environment [3]. Several variables, including microbes, dust, and high temperatures can cause eye illnesses that can result in blindness [4]. Hyperemia/redness, eye discharge, itching, eye pain, and falling lashes are among the most common symptoms in patients with eye infection [5]. The familiar forms of eye infections are conjunctivitis, keratoconjunctivitis, blepharoconjunctivis, keratitis, and ocular trauma [5, 6].

Various infectious agents, such as parasites, bacteria, viruses, and fungi, are responsible for different forms of eye infections [7]. Free-living Acanthamoeba is known to cause keratitis, a rare corneal infection [8, 9] and its incidence has increased over time [10]. Viral infections can cause minor-to-severe ocular discomfort. Many types of eye infections have been linked to Molluscum contagiosum, arboviruses, herpes simplex virus type 1, and common respiratory viruses including adenoviruses [11]. Ocular fungal infections are a significant contributor to ocular morbidity [12]. Aspergillus, Candida, and Fusarium species are the most commonly isolated organisms from ocular specimens [12–14]. Globally, bacterial eye infections in various clinical conditions continue to be a major cause of ocular morbidity and blindness, and their prevalence is periodically increasing [5, 15–17]. Even in developed countries, the prevalence of bacterial isolates from eye specimens is high [12, 18].

Numerous investigations have been conducted in African nations using a variety of ocular specimens from diverse clinical circumstances to ascertain the incidence and type of bacterial infections in the eye [19–21]. A study which was conducted in Ghana indicated 95% prevalence of bacterial isolates, of which the predominant bacterial species were *Pseudomonas aeruginosa* and *Staphylococcus aureus* [5]. A significant proportion of ocular samples were culture-positive among patients from Shashamene, Ethiopia. Furthermore, *S. aureus* and *E. coli* were found to be common isolates in this study [22]. A similar scenario was reported in a study conducted in Nigeria [21].

The rate of antibiotic resistance, particularly multi-drug resistance (MDR), among isolates from eye specimens varies across many studies conducted in developed and developing countries. A study conducted in Taiwan reported low levels of antibiotic resistance to potent drugs [23]. A similar study conducted in developed countries showed a significant level of resistance to amikacin, vancomycin, and moxifloxacin [18]. Similar studies conducted in Ethiopia revealed a substantial level of MDR among bacterial isolates from external eye samples [24, 25].

Empirical therapy, antimicrobial use history, and self-medication practices with broad-spectrum antibiotics are common in Ethiopia and similar regions, influencing the susceptibility profiles of bacterial isolates from ocular infections [24–29]. This study aims to detect bacterial pathogens and assess their susceptibility profiles to commonly used antibiotics in the ophthalmology unit of Boru-meda Hospital, Dessie, Ethiopia.

## Methodology

Study design, setting and population This hospital-based cross-sectional study was conducted from February 1 to April 30, 2021, at Boru-meda General Hospital, which is located in Dessie Town, Northeast Ethiopia. Dessie town was founded in 1882 G. C., and its total area coverage was 15.08 km^2^ (5.82 sq.mi). The town is located in the eastern part of the Amhara Region and in north-central Ethiopia. Dessie is located 401 km north of Addis Ababa. In 1955, Boru-meda General Hospital was founded with the assistance of a missionary organization that provided mostly ophthalmological and dermatological services [30]. Currently, the hospital provides a wide range of services. 2.5 million people in the South and North Wollo catchments, Oromia special zone, and Afar region are served by the hospital. The source population for the study comprised all patients who were receiving care at Boru-meda General Hospital at the time. The study population included patients with symptomatic ocular or peri-ocular infection who visited the Boru-meda General Hospital ophthalmic clinic during the study period. The study included patients who volunteered to provide samples and had symptomatic ocular or peri-ocular infection. However, patients who had received prior ocular surgery within the last seven days, had used an antibiotic ointment for the previous five days, or had taken another drug for the preceding 14 days were not allowed to participate in the study.

### Sample size determination and sampling techniques

The sample size was determined using a single population proportion formula as follows: n = z^2^ p (1-p)/ d^2^, where n is the number of ophthalmic patients to be involved in this study; Z = Standard normal distribution value at 95% CI, which is 1.96; P is the prevalence of bacterial pathogens among ophthalmic patients previously reported in Jimma town, which was 74.7% [31]. P is the prevalence = 0.747; 1-*p* = 0.253; and d is the desired degree of accuracy = 0.05. Therefore, by adding 10% non-response rates, 319 study participants with symptomatic ocular or peri-ocular infected study participants had enrolled using non-probability consecutive sampling technique.

Data Collection and Analysis.

### Specimen collection

The study subjects were examined by an ophthalmologist and a senior ophthalmic nurse using a slit-lamp biomicroscope to screen for the presence of ocular or peri-ocular infection. Ocular specimens were collected from patients with conjunctivitis (*N* = 126), blepharitis (*N* = 47), dacryocystitis (*N* = 45), blepharoconjunctivitis (*N* = 37), keratitis (*N* = 35), trauma (*N* = 15), or endophthalmitis (*N* = 14) [12, 22, 32, 33]. For blepharitis and conjunctivitis, specimens were collected from the eyelids and conjunctiva using a sterile cotton swab moistened with sterile saline by a senior microbiologist and a senior laboratory technologist. The swab was rolled over the eyelid margin and conjunctiva from the medial side to the lateral side and back. For Dacryocystitis cases, pus from the lacrimal sac was collected using a dry sterile cotton-tipped swab either by applying pressure over the lacrimal sac to allow purulent material to reflux through the lacrimal punctum or by irrigating the lacrimal drainage system. In other keratitis cases, corneal scraping using a surgical blade and tearing for ulcerative keratitis were performed by an ophthalmologist or a trained ophthalmic nurse. For endophthalmitis, needle aspiration was performed by a physician or a trained ophthalmic nurse. The samples were immediately inoculated with chocolate agar, blood agar, and MacConkey agar in the microbiology laboratory. Following the specimen collection the socio-demographic information of the study participants was gatherd through face to face interview.

### Cultivation and identification

To enhance the growth of fastidious bacteria, the collected swabs was first inoculated on chocolate agar and 5% sheep blood agar. It was then inoculated onto MacConkey agar for identification of gram-negative bacterial species. For further identification of Staphylococcus species, samples were inoculated on mannitol salt agar (Oxoid, Ltd.) and incubated at 35–37 °C for 24–48 h. Aerobic atmospheric condition was maintained for the MacConkey agar and mannitol salt agar, while whereas chocolate agar and 5% sheep blood agar were incubated a 5–10% CO_2_ atmosphere. All plates were initially examined for growth after 24 h, and cultures with no growth were reincubated for 48 h. If there was no growth after 48 h, the plate was culture-negative. After pure colonies were obtained, identification was performed using standard microbiological techniques including Gram staining, colony morphology, and biochemical tests. Gram-positive bacteria were tested against catalase, DNase and coagulase production. In addition the isolates were studied for mannitol fermentation, optochin and novobicin susceptibility, bile solubility, and hemolysis. On other hand, Gram-negative isolates were examined for sugar fermentation tests on Kliger’s Iron Agar and MacConkey agar. Furthermore gram negative isolates were tested for motility, citrate fermentation, Urease production, and DNase.

### Antimicrobial susceptibility testing

A modified Kirby Bauer disk diffusion method was used to test each isolate for in vitro antimicrobial susceptibility patterns, based on the Clinical and Laboratory Standards Institute (CLSI 2020) criteria. By taking 3–5 freshly grown pure colonies a homogeneous inoculums suspension was prepared using sterile normal saline and adjusted to 0.5 McFarland standards turbidity. A sterile cotton swab was dipped, rotated several times, and pressed against the test tube wall. It was then swabbed over the entire surface of the Mueller Hinton agar (MHA) plates, and for all fastidious or slow-growing bacteria, 5% of sheep blood or chocolate MHA was used. Antimicrobial impregnated paper disks were placed on the plate. The results interpretation of the disk diffusion test are “qualitative,” in that a category of susceptibility (susceptible, intermediate, or resistant) is derived from the test.

### Quality assurance and data analysis

All clinical evaluations were performed by clinicians who were experienced in the care of patients with diseases. The reliability of the cultural results was guaranteed by strict adherence to standard operating procedures (SOPs) and implementation of standard quality control (QC) measures throughout the laboratory process. All the culture plates were prepared according to the manufacturer’s instructions. Sample collection, medium preparation, inoculation, isolation, identification, and antibiotic susceptibility testing were performed under aseptic conditions. The media performance and potency of the antimicrobial discs were tested using the American Type Culture Collection (ATCC) standard reference strains. *E*. *coli (*ATCC 25,922) and *Staphylococcus aureus (*ATCC 25,923) were used. Visual inspection of the media was performed. The sterility of prepared culture plates was assessed by incubating 5% of the batch plate at 35-37^O^C overnight before using it. A 0.5% McFarland turbidity standard was used to standardize bacterial suspensions for the antimicrobial susceptibility testing. Quality control of the Gram stain reagents was checked using known Gram-positive and Gram-negative smears. Data were checked for completeness and coded using Statistical Package for Social Science (SPSS) version 21 for analysis.

## Results

### Socio-demographic characteristics

A total of 319 study participants with a clinical diagnosis of ocular or peri-ocular infection during the study period were included. 109 (34.2%) of the study participants were primarily in the 11–30 years old. Of the participants, 218 (68.3%) lived in rural areas and 182 (57.1%) were married. Information on the participants’ educational background and occupation revealed that 157 (49.2%) and 190 (59.6%) were farmers and illiterates, respectively (Table 1).

**Table 1:** Socio-demographic characteristics (*n* = 319) among patients with ocular or peri-ocular infections at the Eye Unit of Boru-meda General Hospital, February 1 to April 30, 2021

Variables		Frequency	Percent
Age	≤ 10	23	7.2
	11–30	109	34.2
	31–50	96	30.1
	51–70	67	21.0
	≥ 71	24	7.5
Gender	Male	162	50.8
	Female	157	49.2
Residence	Rural	218	68.3
	Urban	101	31.7
Occupation	Student or not applicable	57	17.9
	Farmer	190	59.6
	Merchant	15	4.7
	Gov’t Employed	29	9.1
	House Wife	28	8.8
Marital Status	Married	182	57.1
	Single or not applicable	101	31.7
	Divorce	20	6.3
	Widow	16	5.0
Educational Status	No education.	157	49.2
	Primary School	78	24.5
	Secondary School	52	16.3
	College And Above	32	10.0

### Clinical features and culture positivity rate

The majority of participants developed conjunctivitis 126 (39.5%), followed by blepharitis 47 (14.73%), and dacryocystitis 45 (14.1%). A higher proportion of culture positivity rate was observed among dacryocystitis cases 34 (75.6%), followed by blepharitis 32 (68.1%), blepharo-conjunctivitis 22 (59.5%), and keratitis 16 (45.7%) (Table 2).

**Table 2:** Clinical presentation and culture results among patients with either ocular or peri-ocular infections at the Eye Unit of Boru-meda General Hospital, February 1 to April 30, 2021

Clinical presentation	Culture result		Total, *n* (%)
	Positive, *n* (%)	Negative, *n* (%)	
Conjunctivitis	51(40.5)	75(59.5)	126 (39.5)
Blepharitis	32(68.1)	15(31.9)	47 (14.7)
Dacryocystitis	34(75.6)	11(24.4)	45 (14.1)
Blepharo Conjunctivitis	22(59.5)	15(40.5)	37 (11.6)
Keratitis	16(45.7)	19(54.3)	35 (10.9)
Trauma	4(26.7)	11(73.3)	15 (4.4)
Endophthalmitis	5(35.7)	9(64.3)	14 (4.4)
Total	164(51.4)	155(48.6)	319 (100)

### Prevalence and profile of bacterial isolates

Of 319 participants with suspected ocular or peri-ocular bacterial infections, 164 (51.4%) were culture positive. The majority of the patients 158 (49.5%) had single bacterial isolates, whereas only 6 (1.9%) participants had mixed bacterial isolates. Nighty five (29.8%) participants had Gram-positive bacterial infections with one mixed infection, and 69 (21.6%) had Gram-negative bacterial infections with five mixed infections, giving a total of 170 (53.3%) bacterial isolates. The proportion of Gram-positive and Gram-negative isolates was 96 (56.5%) and 74 (43.5%), respectively. The predominant species was CoNS 47 (27.6%), followed by *S. aureus* 38 (22.4%) and *Moraxella species* 32 (18.8%) (Table 3).

**Table 3:** Types of bacteria isolated and clinical presentation of participants with either ocular or peri-ocular infections at Eye Unit of Boru-meda General Hospital, February 1 to April 30, 2021 (*N* = 319)

Bacterial species	Clinical presentation and their frequency							
	Dacryocystitis *N* = 45 *n* = 35	Blepharitis, *N* = 47, *n* = 35	Conjunctivitis *N* = 126 *n* = 52	Blephero-Conjunctivitis, *N* = 37 *n* = 23	Trauma, *N* = 15 *n* = 4	Keratitis, *N* = 35 *n* = 16	Endophtalmitis *N* = 14 *n* = 5	Total isolates *N* = 170
**Gram negative**	**n (%)**	**n(%)**	**n (%)**	**n (%)**	**n (%)**	**n (%)**	**n (%)**	**n (%)**
*Moraxella species(n = 32)*	13 (37)	8(22.9)	5(9.6)	6(26.1)		-	-	32(18.8)
*H. influenzae*(n = 9)	2(5.7)	1(2.9)	3(5.7)	-	-	3(5.7)	-	9(5.3)
*N. gonorrhoeae*(n = 7)	4(11.5)		2(3.9)	1(4.3)	-	-	-	7(4.11)
*P. aeruginosa*(n = 3)	-	-	1(1.9)	1(1.9)	-	1(1.9)	-	3(1.8)
*E. coli* (n = 5)	1(2.9)	-	3(5.7)	1(4.6)	-	-	-	5(2.9)
*Acinetobacter species*(n = 5)	2(5.7)	-	2(3.9)	1(4.3)	-	-	-	5(2.9)
*Shigella species (n = 4)*	2(5.7)	2(5.7)	-	-	-	-	-	4(2.4)
*Other species* (n = 9)	2(5.7)	2(5.7)	4(7.7)	-	-	-	1 (20)	9(5.3)
Total (*n* = 74)	26(74.3)	13(37.1)	20(38.5)	10(43.5)	0(0)	4 (25)	1 (20)	74(43.5)
**Gram-positive**								
*S. aureus* (n = 38)	3(8.9)	10(28.6)	7(13.5)	6(26.1)	1 (25)	7(43.8)	4(80)	38(22.4)
*CoNS (n = 47)*	0	10(28.6)	24(46.2)	6(26.1)	3(75)	4 (25)		47(27.6)
*S. pneumonia (n = 6)*	4(11.4)		1(2.9)	1(4.3)				6(3.5)
*group D viridian Streptococcus species (n = 5)*	2(5.7)	2(5.7)				1(6.25)		5(2.9)
Total (*n* = 96)	9(25.7)	22(62.9)	32(61.5)	13(56.5)	4(100)	12(75)	4(80)	96 (56.5)
Isolation rate	35(77.8)	35(74.5)	52(41.3)	23(62.2)	4(26.7)	16(45.7)	5(35.7)	170 (53.3)

**NOTE**: N = number of clinical case; n = total number of bacteria found in clinical case; CoNS = Coagulase Negative Staphylococcus; Other species including *Klebsiella oxytoca* (*n* = 2), *Klebsiella ozaenae* (*n* = 1), *Proteus mirabilis* (*n* = 2), *Citrobacter species* (*n* = 1), *Providencia rettgeri* (*n* = 1), *Morganella morganii* (*n* = 2)

### Antimicrobial susceptibility profile of Gram negative isolates

The majority of isolated gram negative bacteria were resistant to tetracycline, 28 (71.8%). Moraxella species showed a substantial level of susceptibility to certain antimicrobials such as amoxicillin-clavulanate, gentamicin, and ciprofloxacin, but the isolates demonstrated a significant level of resistance to tobramycin. *N. gonorhoea* isolates showed high levels of resistance to penicillin and tetracycline, and moderate to high levels of susceptibility to ciprofloxacin and ceftriaxone, respectively (Table 4).

**Table 4:** Antimicrobial susceptibility profile of Gram-negative bacteria isolated from patients with either peri-ocular or ocular infection at Eye Unit Boru-meda General Hospital, February1to April 30, 2021

Antimicrobials	Isolated bacterial spp and their AST pattern															
	Moraxella spps. *n* = 32		*H.influnzae, n = 9*		Neisseria gonorrhoeae *n* = 7		*P*. aeruginosa *n* = 3		E.coli *n* = 5		Acinetobacter species *n* = 5		Shigella *species n = 4*		Total Gram negative *n* (%)	
	S	*R*	S	*R*	S	*R*	S	*R*	S	*R*	S	*R*	S	*R*	S	*R*
P	13(40.6)	19(59.4)	ND	ND	1(14.3)	6(85.7)	ND	ND	ND	ND	ND	ND	ND	ND	14(35.9)	25(64.1)
TE	ND	ND	1(11.1)	8(88.9)	-	7(100)	ND	ND	3(60)	2 (40)	2 (40)	3(60	2 (50)	2 (50)	11(28.2)	28(71.8)
ERY	ND	ND	ND	ND	ND	ND	ND	ND	4(80)	1 (20)	ND	ND	2 (50)	2(50)	7(38.9)	11(61.1)
AZM	ND	ND	8(88.9)	1(11.10	ND	ND	ND	ND	4(80)	1 (20)	ND	ND	2 (50)	2 (50)	15(55.6)	12(44.4)
CPR	32(100)	-	9(100)	-	5(71.4)	2(28.6)	3(100)	-	5(100)	-	4(80)	1 (20)	4(100)	-	68(73.3)	6(25.7)
DOX	ND	ND	3 (33.3)	6(66.7)	ND	ND	ND	ND	-	5(100)	3(60)	2 (40)	2 (50)	2 (50)	10(31.3)	22(68.7)
C	ND	ND	3(33.30	6(66.7)	ND	ND	ND	ND	2 (40)	3(60)	ND	ND	2 (50)	2 (50)	13 (48)	14 (52)
GEN	28(87.5)	4(12.5)	ND	ND	ND	ND	3(100)	-	5(100)	-	5(100)	-	4(100)	-	54(93)	4 (7)
COT	ND	ND	3(33.3)	6(66.7)	ND	ND	ND	ND	ND	ND	3(60)	2 (40)	ND	ND	6(42.9)	8(57.1)
NAL	ND	ND	ND	ND	ND	ND	ND	ND	5(100)	-	ND	ND	4(100)	-	17(94.4)	1(5.6)
AUG	31(96.9)	1(3.1)	9(100)	-	ND	ND	ND	ND	5(100)	-	ND	ND	4(100)	-	53(89.8)	6(10.2)
AMP	ND	ND	-	9(100)	ND	ND	ND	ND	-	5(100)	ND	ND	-	4(100)	0	27(100)
PIF	ND	ND	ND	ND	ND	ND	2(66.7)	1(33.3)	4(80)	1 (20)	4(80)	1 (25)	3(75)	1 (25)	20(76.9)	6(23.1)
TOB	6(18.8)	26(81)	ND	ND	ND	ND	-	3(100)	ND	ND	ND	ND	ND	ND	6(17.1)	29(82.9)
CTR	ND	ND	9(100)	-	6(85.7)	1(14.3)	2(66.7)	1(33.3)	5(100)	-	ND	ND	4(100)	-	30(81.1)	7(18.9
VAN	32(100)	-	ND	ND	ND	ND	ND	ND	ND	ND	ND	ND	ND	ND	32(100)	0

**NOTE**: S-susceptible, R-resistant, P-Penicillin, TE-tetracycline, ERY-erythromycin, AZM-azithromycin, CPR-ciprofloxacin, DOX-doxycycline, C-chloramphenicol, GEN-gentamicin, COT-trimethoprim/sulphamethaxzole, NAL nalidixic acid, AUG-amoxicillin- clavulanate, AMP-ampicillin, PIF-piperacillin, TOB-tobramycin, CTR-ceftriaxone and VAN-vancomycin, ND-Not done, Other spp includes *Klebsiella oxytocin* (*n* = 2), *Klebsiella ozanae* (*n* = 1), *Proteus mirabilus* (*n* = 2), *Citrobacter spp* (*n* = 1), *Providencia rettegri* (*n* = 1), *Morganella morganai* (*n* = 2)

### Antimicrobial susceptibility profile of Gram positive isolates

Gram-positive bacteria exhibit substantial sensitivity to nitrofurantoin, clindamycin, gentamycin, ceftriaxone, and vancomycin. Most *S. aureus* isolates were found to be susceptible to azithromycin, ciprofloxacin, clindamycin, gentamicin, and nitrofurantoin. All S. *aureus isolates* were resistant to penicillin and approximately 32 (84%), 33 (86.3%), 20 (52.6%), and 18(47.4) isolates had demonstrated resistant to trimethoprim/sulfamethoxazole, tetracycline, doxycycline, and erythromycin, respectively. Similarly, 47 (100%), 44 (93.6%), 40 (85.1%), and 39 (82.9%) *CoNS* isolates were susceptible to nitrofurantoin, clindamycin, gentamicin, and ciprofloxacin, respectively. However, a significant proportion of *CoNS* isolates showed resistance to penicillin, trimethoprim/sulfamethoxazole, tetracycline, and erythromycin. All *S. pneumoniae* isolates were susceptible to vancomycin, but resistant to tetracycline and meropenem (Table 5).

**Table 5:** Antimicrobial susceptibility pattern of Gram-positive bacteria isolated from patients with either peri-ocular or ocular infection at Eye Unit Boru-meda General Hospital, February 1 to April 30, 2021 (*n* = 96)

Antimicrobials	Isolated bacterial species and their AST pattern										
	*S. aureus* n = 38		*CoNS n = 47*		*S. pneumoniae n = 6*		*Group-d-viridian streptococci n = 5*		Gram positive Total n(%) *n* = 96		
	S; *n* (%)	*R*; *n* (%)	S; *n* (%)	*R*; *n* (%)	S; *n* (%)	*R*; *n* (%)	S; *n* (%)	*R*; *n* (%)	*n* (%)S	*n* (%)*R*	
P	-	38(100)	1(2.1)	46(97.9)	1(16.7)	5(83.3)	1 (20)	4(80)	3(3.125)	93(96.9)	
TE	5 (13.2)	33(86.3)	10(21.3)	37(78.7)	-	6(100)	1 (20)	4(80)	16(16.7)	80(83.3)	
ERY	20(52.6)	18(47.4)	12(25.5)	35(74.5)	3 (50)	3 (50)	2 (40)	3(60)	37(38.5)	59(61.5)	
AZM	31(81.6)	7(18.4)	23(48.9)	24(51.1)	5(83.3)	1(16.7)	5(100)	-	64(66.7)	32(33.3)	
CPR	36(94.7)	2(5.4)	39(82.9)	8(17.1)	6(100)	-	5(100)	-	86(89.6)	10(10.4)	
DOX	18(47.4)	20(52.6)	20(42.6)	27(57.4)	1(16.7)	5(83.3)	1 20)	4(80)	40(41.7)	56(58.3)	
CLN	36(94.7)	2(5.3)	44(93.6)	3(6.4)	6(100)	-	5(100)	-	91(94.8)	5(5.2)	
C	25(65.8)	13(34.2)	28(59.6)	19(40.4)	3 (50)	3 (50)	2 (40)	3(60)	58(60.4)	38(39.6)	
GEN	36(94.7)	2(5.3)	40(85.1)	7(14.9)	6 (100)	-	5 (100)	-	87(90.6)	9(9.4)	
SXT	6 (15.8)	32(84.2)	3 (6.4)	44(93.6)	1 (16.7)	5(83.3	-	5(100	10(10.4)	86(89.6)	
NIT	38(100)	-	47(100)	-	ND	ND	ND	ND	85(100)	0	
MER	ND	ND	ND	ND	-	6(100)	-	5(100)	0	11(100)	
IMP	ND	ND	ND	ND	6 (100)	-	5(100)	-	11(100)	0	
CTR	ND	ND	ND	ND	6 (100)	-	4 (80)	1 (20)	10(90.9)	1(9.1)	
VAN	ND	ND	ND	ND	6 (100)	-	5(100)	-	11(100)	0	

**NOTE**: S-susceptible, R-resistant, P-penicillin, TE-tetracycline, ERY-erythromycin, AZM-azithromycin, CPR-ciprofloxacin, DOX-doxycycline, CLN-clindamycin, C-chloramphenicol, GEN-gentamicin, SXT-trimethoprim-sulfamethoxazole, NIT-nitrofurantoin, MER-meropenem, IMP-impenem, CTR-ceftriaxone, and VAN-vancomycin, ND-Not done

### Multi-drug resistance

Thirty-six (21.2%) bacterial isolates were resistant to two antimicrobial drugs, while seven (4.1%) of the isolates were sensitive to all of the tested antimicrobial agents. The overall MDR rate was 62.9%; 33 (44.6%) of the gram-negative isolates and 74 (77.1%) of the gram-positive isolates were confirmed to be MDR. 37 (78.7%) and 29 (76.3%) of all the CoNS and *S. aureus* isolates were MDR, respectively (Table 6).

**Table 6:** Level of anti-microbial resistance isolated from peri-ocular and ocular infection at Eye Unit of Boru-meda General Hospital, February1 to April 30,2021 (*n* = 170)

Bacterial isolate		Level of resistance pattern							
		Ro	R1	R2	R3	R4	R5	R6	R7
Gram positive	*S. aureus (n = 38)*	1(3.8)	1(3.8)	7(18.4)	10(26.3)	13(34.2)	1(3.8)	2(5.3)	3(7.9)
	*CoNS (n = 47)*	1(2.1)	1(2.1)	8 (17)	8 (17)	9(19.1)	7(14.9)	6(12.8)	7(14.9)
	*S. pneumoniae (n = 6)*	0	2(33.3)	0	0	1(16.7)	1(16.7)	0	2(33.3)
	*Group-D-Streptococci(n = 5)*	1 (20)	0	0	0	1 (20)	2 (40)	0	1 (20)
	Total Gram positive (*n* = 96)	3(3.1)	4(4.2)	15(15.6)	18(18.8)	24 (25)	11(11.5)	8(8.3)	13(13.50
Gram negative	*Moraxella spp (n = 32)*	4(12.5)	11(34.4)	12(37.5)	5(15.6)	0	0	0	0
	*H. influenzae (n = 9)*	0	2(22.2)	2(22.2)	1(11.1)	2(22.2)	1(11.1)	1(11.1)	0
	*Neisseria gonorrhoeae (n = 7)*	0	2(28.6)	1(14.3)	3(42.9)	0	1(14.3)	0	0
	*Pseudomonas species (n = 3)*	0	0	2(66.7)	1(33.3)	0	0	0	0
	*K. oxytoca (n = 2)*	0	0	0	0	0	0	0	2(100)
	*E. coli (n = 5)*	0	0	0	2 (40)	2 (40)	1 (20)	0	0
	*Acinetobacter species (n = 5)*	0	0	2 (40)	1 (20)	2 (40)	0	0	0
	*Proteus Mirabilis(n = 2)*	0	0	0	0	0	0	0	2(100)
	*Shigella species (n = 4)*	0	0	1 (25)	1 (25)	0	1 (25)	0	1 (25)
	*Citrobacter species(n = 1)*	0	0	0	0	0	0	0	1(100)
	*P. rettgeri (n = 1)*	0	0	1(100)	0	0	0	0	0
	*M. morganii (n = 2)*	0	0	0	0	0	1 (50)	0	1 (50)
	*K. ozaenae (n = 1)*	0	1(100)	0	0	0	0	0	0
	Total (*n* = 74)	4(5.4)	16(21.6)	21(28.4)	14(18.9)	6(8.1)	5(6.8)	1(1.4)	7(9.5)
Overall (*n* = 170)		7(4.1)	20(11.8)	36(21.2)	32(18.8)	30(17.6)	16(9.4)	9(5.3)	20(11.8)

**Note**: R0 Susceptible for all antimicrobial, R1 resistant for one antimicrobial, R2 resistant for 2 antimicrobials, R3 resistant for 3 antimicrobial, R4 resistant for 4 antimicrobial, R5 resistant for 5 antimicrobial, R6 resistant for 6 antimicrobial, R7 resistant for 7 and above antimicrobial

## Discussion

In the current study, we examined the bacterial profile of peri-ocular or ocular infections in patients of all ages enrolled at the Boru-meda General Hospital. The prevalence of at least one bacterial infection in the current study was 51.4%, with a confidence interval of 95% CI (46.1–56.7%). The findings of this study are comparable to those of other studies conducted in Ethiopia [34], India [35], and Egypt [36]. The prevalence of the present study was lower than previous reports from various parts of Ethiopia such as Tigray (66.7%) [17] Jimma (74.7%) [31], Shashmene (59.6%) [22], Addis Ababa (59.4%) [37] and Gondar (58.3%) [25]. Similarly, it was lower than studies conducted in other countries such as Sudan (78%) [38], Nigeria (67.28%) [20], Uganda (59.5%) [19], and Iran (75.2%) [39]. However, the current findings are higher than those of previous reports from India [40] and China [41]. This difference may be attributed to variations in the study period, population, and geographic location.

Among all bacterial isolates, gram-positive bacteria contributed to the majority (56.5%) of the isolates, with a confidence interval of 95% CI (48.8–64.1%). This finding is comparable to previous reports from Tigray (60.7%) [17] and Iran (61.8%) [39]. However the finding was low as compared to previous reported from Ethiopia such as studies conducted in Shashmene [22], Addis Ababa Ethiopia [37, 42], Jimma [31], Gondar [25] and Jigjiga [43]. It was also lower than that reported in similar studies conducted in other countries such as Nigeria [20], Italy [44] and China [41]. Overall, the predominant gram-positive bacterial isolate was *CoNS* (27.6%) the same as previously reported for Addis Ababa (31.6%) [37], Gondar (33.5%) [25] and Jimma (27.7%) [34]. On the other hand, it was lower than those reported in Sudan (41%) [38], Egypt (48%) [36], and the USA (54%) [7].

The second predominant bacterial isolate in the current study *was S. aureus* which accounted for 22.4% and the finding was in agreement with previous study conducted in Ethiopia [17]. It was also comparable to studies conducted outside Ethiopia, such as Iran [39], Uganda [19] and the USA [7]. However, in the current study, the number of *S. aureus* isolates was lower than that reported in other studies conducted in Addis Ababa [37, 42], Jimma [31], Gondar [25, 45] and other countries such as Sudan [38], Spain [46] and India [35]. The predominance of Gram-positive cocci might be due to variation among the study population and might also be due to contamination of the injured eye from skin floras. However, virulence factors, microbial adherence (fibronectin), and defects in host defence mechanisms may increase this infection. It is possible that the lower rate of *S. aureus* in this study is because some bacterial carbohydrates cause an inflammatory reaction on their surface, which can lower the bacterial burden, but also cause tissue injury. Tissue damage is mediated by secreted proteins that aid the activation of the inflammatory response. Examples of these proteins include alpha-, beta-, gamma-, and Panton-Valentine leucocidin [47].

The proportion of gram-negative bacteria isolates was 43.5%, with a 95% CI (35.9–51.2%). This is in agreement with a previous studies conducted in Tigray (39.3%) [17], Jimma (48%) [31], and Iran (39%) [39]. This was higher compared to previous reports from Ethiopia, like studies conducted in Shashmene (31.8%) [22], Addis Ababa (25.4–30%) [37, 42], Gondar (22–31.6%) [25, 45] and some other countries, such as Sudan (26.9%) [38], Nigeria (31%) [20] and China (30%) [41]. Changes in the normal flora, hygienic settings, virulence factors, microbial adhesion, and flaws in host defense mechanisms can all contribute to an increase in the number of gram-negative bacteria. Another potential explanation for the variation in the percentage of gram-negative isolates across studies could be the study population, duration of investigation, laboratory configuration, and other circumstances.

Among Gram-negative bacterial isolates in the current study, *Moraxella species* (18.8%) was the most prevalent, followed by *H. influenzae* (5.3%) and *N. gonorrhoeae* (4.11%), respectively. The prevalence of *Moraxella species* was higher than those reported in studies conducted in Ethiopia [22, 37, 43] and Italy [29]. On the other hand prevalence of *H. influenza* was higher than reported in studies conducted in Tigray (1.1%) [17], Shashmene (3%) [22], Jimma (3.4%) [34], and other countries like Nigeria (1.38%) [20] and Sudan (2.6%) [38]. As compared with the USA (9%) [7] and Iran (11.4%) [39], the prevalence of *H. influenzae* in the current study was low.

The fact that Moraxella species are commensals in the mucosal membranes of the mouth, upper respiratory tract, and genitourinary tract may be the cause for the increase in Moraxella species. Individuals with impaired nutritional status, immune system weakness, persistent corneal epithelial abnormalities (previous ocular disease), and increased production of endotoxins and proteases can have an increased risk of eye infection [48]. Similar to the Moraxella species, the nasopharynx is the source of most eye infections caused by *H. influenzae*. The organism generates an IgA protease that facilitates adhesion to the ocular mucosa by breaking down the secretory IgA [49].

The detection of Shigella species in the present study (4; 2.4%) might be linked with the socio-demographic features of the study participants, like age, residence, occupation and educational status. Shigella species are known to cause shigellosis (bacillary dysentery) which is feaco-orally transmitted disease and there might be the possibility to infect the eye of an individual who did not have good personal hygiene practices. According to our report, it is possible to understand the need for further studies regarding the detection of Shigella species from ocular site.

In our study, most of CoNS isolates were resistant to (97.9%) penicillin, (78.7%) tetracycline, and (74.5%) erythromycin. This finding is consistent with those of other studies conducted in Ethiopia [22, 37, 42]. However, in Uganda, CoNS showed lower resistance rate to tetracycline and erythromycin which is not in agreement with the present study [19]. The majority of *S. aureus* were found to be resistant to (86.3%) tetracycline, (100%) penicillin, (47.4%) erythromycin, (84.2%) trimethoprim/sulfamethoxazole; this finding in the current study was comparable with some studies conducted in various areas of Ethiopia [17, 22, 37, 42, 45]. However, the susceptibility pattern of the species to trimethoprim/sulfamethoxazole and erythromycin was found to vary in comparison to a study conducted in Gondor [25]. This variation might be due to genetic variations in which the bacteria can develop mutations, as it is known that mutations in bacterial DNA can render antibiotics ineffective. The highest resistance of *S. aureus* and *CoNS* to penicillin, as presented in the current study, might be due to the production of beta-lactamase enzymes and alteration of penicillin-binding proteins. In the present study, *S. pneumonia* isolates were resistant to doxycycline and meropenem. Some studies have indicated that the emergence of meropenem-resistant clones (strains) of pneumococcus, such as 15 A-ST63, is strongly associated with resistance to meropenem drug [50–52]. In contrast to the current study, a study conducted in Jigjiga, Ethiopia indicated that the isolate was highly susceptible to doxycycline [43].

In the current study, the prevalence of multidrug resistance was 62.8%, which is in agreement with a study conducted in Debre-Markos, Ethiopia [53]. The rate of MDR in the present study was higher than studies conducted in the Ethiopia region (53%), Tigray [17], and China (12.1%) [41] but it was lower than previous studies done in (87%), Gondar [25] and (71.2%) Addis Ababa [42]. This may be due to the irrational use of antimicrobials without prescription, improper dosage regimen, misuse of antimicrobials for viral and other non-bacterial infections, extended duration of therapy, biofilm formation, transfer of resistance plasmid genes, efflux, and production of extended-spectrum β-lactamases, which increase anti-microbial resistance.

### Limitation of the study

A limitation of the current study was that bacteria *Chlamydia trachomatits*, anaerobic bacteria, fungal species, viral agents, and parasitic species that can cause ocular infections were not investigated owing to resource and laboratory infrastructure limitations. In addition, this study was conducted at a single healthcare institute that may not accurately represent the entire catchment area around the southern wollo zone. The recommended strains for chocolate agar media quality control are *N. gonorrhoeae* ATCC 49,226 and *H. influenzae* ATCC 49,766. But due to unavailability of the strains during the study period we did not use the strains and this might affect the research output to some extent.

## Conclusion

In the present study, conjunctivitis was the most common clinical case, followed by blepharitis and dacryocystitis. *CoNS, S. aureus, and Moraxella species* were predominant isolates. The majority of the gram-positive bacterial isolates were multidrug-resistant. *CoNS* and *S. aureus* were the most antibiotic-resistant species among gram-positive isolates. A higher rate of MDR has been observed in ocular and peri-ocular infections. Efficient peri-ocular or ocular bacterial surveillance, including microbiological laboratory data, is necessary to monitor disease trends and risk groups. A large-scale nationwide prospective study is required to achieve this goal.

### Electronic supplementary material

Below is the link to the electronic supplementary material.

Supplementary Material 1

## Data Availability

Those who want to find the original data used for this study are available at the corresponding author, so interested readers can obtain the data from the corresponding author upon reasonable request.

## Abbreviations

ATCC: American Type Culture Collection
MDR: Multi-Drug Resistance
CoNS: Coagulase Negative Staphylococcus
MHA: Mueller-Hinton agar
SOPs: Standard Operating Procedures
